# Opioid sparing anesthesia in patients with liver cirrhosis undergoing liver resection: a controlled randomized double-blind study

**DOI:** 10.1186/s12871-025-02915-4

**Published:** 2025-02-01

**Authors:** Eman Sayed Ibrahim, Ahmed A. Metwally, Mohamed Abdullatif, Essam A. Eid, Mahmoud G. Mousa, Amany A. Sultan

**Affiliations:** 1 Department of Anaesthesiology, Intensive Care and Pain Management, National Liver Institute – Menoufia University, Menoufia, Egypt; 2 Department of Anaesthesiology, Intensive Care and Pain Management, Faculty of Medicine, Menoufia University, Menoufia, Egypt; 3 Department of Anaesthesiology and Surgical Intensive Care, Faculty of Medicine, Cairo University, Giza, Egypt; 4 Intensive Care and Pain Management, National Liver Institute – Menoufia University, Menoufia, Egypt; 5 5 Abdullah Ismael-Nasser EL thawra EL haram, Giza, Egypt

**Keywords:** OSA, Liver cirrhosis, Liver resection

## Abstract

**Objective:**

Opioid metabolism and pharmacodynamics may be affected in hepatic patients. Ketamine and dexmedetomidine are conventional anesthetics used in our daily practice. The opioid-sparing effects of this combination have not been evaluated in patients with liver cirrhosis undergoing liver resection. We aimed to investigate the potential peri-operative opioid-sparing effects of intra-operative dexmedetomidine and ketamine infusions in patients with Child A liver cirrhosis undergoing liver resection.

**Methods:**

This study was a randomized controlled double-blind trial. 92 adult patients of both sex with Child class (A) liver cirrhosis aged 18 to 65 years entering and completing the study. We excluded patients with renal or cardiac dysfunction or contraindications from study medications.46 patients in the opioid-sparing group (OS) receiving ketamine and dexmedetomidine infusions and 46 patients in the opioid-based (OB) group as controls. The main outcome measures: were intra-operative fentanyl requirements, postoperative fentanyl requirements, visual analogue pain scores, postoperative nausea, vomiting, ileus, desaturation, intra-operative hemodynamic events, and ICU stay were recorded.

**Results:**

The total intra-operative fentanyl consumption was significantly lower in the OS group compared with the OB group, 183.2 ± 35.61 µg and 313.5 ± 75.06 µg, respectively, *P* < 0.001. The postoperative 1st 48 h fentanyl consumption was significantly lower in the OS group compared with the OB group, 354.5 ± 112.62 µg and 779.1 ± 294.97 ± µg, respectively, *P* < 0.001. Visual analogue scores were significantly better in the OS group at the early 2-hour assessment point postoperatively. The postoperative adverse events were significantly more frequent in the opioid-based group. ICU stay was significantly shorter in the OS group.

**Conclusions:**

Administering dexmedetomidine and ketamine infusions intra-operatively to patients with Child A liver cirrhosis undergoing liver resection resulted in notable opioid-sparing effects, with reductions of approximately 40% intra-operatively and 55% postoperatively. The opioid-sparing group exhibited improved postoperative outcomes, including reduced pain, decreased incidence of opioid-related side effects and shorter ICU stays.

**Supplementary Information:**

The online version contains supplementary material available at 10.1186/s12871-025-02915-4.

## Introduction

The introduction of the balanced general anaesthesia concept using different medications for each desired effect, as first explained by Cecil Gray in 1946, was a turning point after 100 years of inhalation anaesthesia [1]. The opioid components in the balanced general anaesthesia triad reduced the dose and side effects of anaesthetic and hypnotic medications [2]. Nevertheless, opioids have many side effects including postoperative nausea and vomiting, hyperalgesia, postoperative respiratory depression [3, 4], and possible immunosuppression [5, 6]. For enhanced recovery after surgery, it is recommended to reduce the use of perioperative opioids to a minimum or zero whenever possible (Opioid Free Anaesthesia) [7]. 

Opioid metabolism and pharmacodynamics may be affected in patients with hepatic dysfunction either due to a defect in hepatic biotransformation or reduced protein binding [8]. Therefore, in patients with liver cirrhosis, opioids should be administrated with a longer interval between doses and possibly at lower doses [9]. 

Opioid-sparing anaesthesia is a technique where minimal intraoperative opioid is administered. Opioid-sparing anaesthesia is usually achieved through sympatholysis, analgesia, and anaesthesia with dexmedetomidine and analgesia with low-dose ketamine. In addition, paracetamol, magnesium sulphate, lidocaine infusion, and non-steroidal anti-inflammatory drugs (NSAIDs) may be used as adjuncts to the multi-modal opioid-sparing regimen [10]. 

Dexmedetomidine is a highly selective alpha-2 adrenergic receptor agonist that has sedative, analgesic, and opioid-sparing properties [11]. Dexmedetomidine was shown to reduce postoperative pain scores, perioperative opioid consumption, and accordingly, opioid-related side effects [12, 13]. The use of dexmedetomidine for anaesthesia in patients with liver cirrhosis can improve hemodynamic stability, reduce stress response and reduce inflammatory response without affecting immune function [14]. 

Ketamine, an N-methyl-d-aspartate (NMDA) antagonist, blunts central pain sensitization at sub-anaesthetic doses and has been studied extensively as an adjunct for perioperative analgesia. Sub-anaesthetic ketamine doses improve pain scores and reduce perioperative opioid consumption in a broad range of surgical procedures [15]. 

While ketamine and dexmedetomidine are conventional anesthetics used in our daily practice. To the best of our knowledge, the opioid-sparing effects of this combination have not been evaluated in patients with liver cirrhosis undergoing liver resection. This study aimed to investigate the possible opioid-sparing effects and major perioperative events associated with this combination in this patient population.

## Materials and methods

### Inclusion and exclusion criteria

Ethical approval for this study (Ethical Committee N° 00309/2022) was provided by the Institutional Review Board of the National Liver Institute, Menoufia University (Chairperson Prof Azza. Abd Elaziz) on 31 July 2022. The study was registered in the ClinicalTrials.gov identifier number: NCT05674877. The study was conducted in the Anesthesiology Department, National Liver Institute between August 2022 and July 2023. We confirm that all experiments were performed in accordance with relevant guidelines and regulations. Written informed ethics approval and consent to participate was taken from each patient. The study included adult patients with Child class (A) liver cirrhosis aged 18 to 65 years undergoing liver resection. We excluded patients with renal or cardiac dysfunction (calculated GFR by Cockcroft-Gault equation less than 60 ml/min), (Revised cardiac risk index: RCRI score above 2), a history of chronic pain, alcohol or drug abuse, analgesic use in the last 24 h before surgery, major intraoperative hemodynamic instability(If vasoactive agents infusion needed to maintain stable haemodynamic), the need for postoperative ventilation, inability to comprehend pain assessment and allergy or contraindication to any of the study medications.

### Randomization and blindness

Patients were randomly allocated into one of the two study groups: the opioid-sparing group (OS) and the opioid-based group (OB). The randomization was conducted using a computer-generated randomization sequence from an online program (http://www.randomizer.org). The randomization process was performed with a 1:1 allocation ratio to ensure equal distribution of participants between the two groups.

Allocation numbers were concealed in opaque, sealed envelopes. These envelopes were prepared in advance and stored securely until the moment of assignment. Each envelope contained the group assignment corresponding to the randomization number, and the envelope was only opened immediately before surgical intervention. Blindness was maintained for the patients and the attending anaesthesiologist.

### Anesthesia technique

Patients fulfilling the inclusion criteria underwent clinical evaluation including preoperative laboratory assessment of liver and renal functions the day before surgery. The preoperative assessment was conducted according to the guidelines from the European Society of Anesthesiology and Intensive Care. Other diagnostic or laboratory workup was requested by the attending anesthesiologist and the surgeon according to the patient clinical condition.

Basic intraoperative monitoring included: electrocardiography, pulse oximetry, end-tidal CO_2_, invasive arterial blood pressure, central venous pressure, electrical cardiometry (EC) (ICON monitor; Cardiotronics Inc., La Jolla, CA, USA), esophageal temperature, fraction inspired oxygen, expired end-tidal desflurane concentration, and urine output. Depth of anaesthesia was monitored using Bispectral index, and neuromuscular function with TOF-Watch SX (Schering-Plough, Swords, Co. Dublin, Ireland).

The success and spread of the ultrasound-guided bilateral transversus abdominis plane (TAP) blocks were assessed in all patients by the pinprick test after administering 20 ml of 0.25% levobupivacaine on each side before induction of anaesthesia. Anaesthesia was induced in all patients using fentanyl 2 µg kg^− 1^, propofol 2 mg kg^− 1^, and rocuronium 0.6 mg kg^− 1^ to facilitate endotracheal intubation.

### Interventions

After induction of anaesthesia, patients were randomly allocated in two groups opioid sparing group (OS) and opioid-based group (OB). Patients in the opioid-sparing group received a loading dose of dexmedetomidine (1 µg kg^− 1^ over 10 min). This was followed by a fixed continuous maintenance infusion of 0.5 µg kg^− 1^h^− 1^. Furthermore, a single induction analgesic dose of 0.5 mg kg^− 1^ ketamine was given to all patients in the OS group. This was followed by 0.25 mg kg^− 1^ h^− 1^ continuous maintenance infusion. Dexmedetomidine and ketamine infusions were stopped 30 min before the conclusion of surgery. Patients in the opioid-based group received placebo-equivalent boluses and infusions of 0.9% saline. The attending anesthesiologist was blinded to the patient group assignment.

Anaesthesia was maintained with air, oxygen, and desflurane to keep a BIS value between 40 and 60. Muscle relaxation was maintained by additional top-up doses of rocuronium.

0.15 mg kg^− 1^ and was guided by the response to ulnar nerve stimulation. Ventilation parameters were adjusted to maintain normocapnia. Intraoperative normothermia was maintained using a forced air warm blanket (Model 750-Bair Hugger Temperature Management Unit, SMA MISR, Arizant Healthcare Inc, USA), a humidifier, and warm intravenous fluids. Deep venous thrombosis (DVT) prophylaxis included elastic stockings and, sequential compression device (SCD) (Kendall Company, Tyco, USA) on the lower limb until early ambulation. Intraoperative fluid, fresh frozen plasma, and blood replacement therapy were guided by the continuous monitoring of the central venous pressure and EC Cardiometry and were titrated to maintain hemodynamic stability and a hemoglobin level of 10 g dL^− 1^.

The intra-operative hemodynamic target was the mean arterial blood pressure and heart rate within 20% of the baseline value. Significant hemodynamic alterations were managed as follows: bradycardia (heart rate < 50 beats min^− 1^) was managed by incremental 0.5 mg doses of atropine, hypertension and or tachycardia defined as more than 20% increase of the baseline readings was managed by top-up doses of fentanyl 1 µg kg^− 1^ in the two study groups, hypotension defined as more than 20% reduction in the baseline mean arterial blood pressure was managed by incremental doses of ephedrine 5 mg in the two study groups.

At the end of surgery and when two responses to train-of-four ulnar nerve stimulation were detected (T2), residual rocuronium-induced neuromuscular block was antagonized by sugammadex 2 mg kg-1. Patients were extubated and discharged to the surgical intensive care unit after achieving a train-of-four ratio of 0.9. Postoperative analgesia was achieved using patient-controlled fentanyl infusion (PCA fentanyl). Patients were discharged to the surgical ICU overseen by the anaesthesia team at our institution, whereby the postoperative outcomes were evaluated by the attending anaesthetist who was blinded to the patient’s assigned group.

### Study outcomes

The Primary outcome measure was the Intra-operative fentanyl requirements. The Secondary outcome measures included: (1) postoperative PCA fentanyl requirements over the first 48 h postoperatively; (2) incidence of severe postoperative opioid-related adverse events as desaturation episodes (on room air), postoperative nausea and vomiting and postoperative ileus [time Frame: 48 h after extubation. Desaturation is a decrease of oxygen saturation equal to or exceeding 4% of the baseline value. Postoperative ileus is defined as the absence of flatus or stools within the first 48 h after extubation; (3) incidence of bradycardia, hypotension, and hypertension events during surgery and the number and doses of rescue medications during surgery (fentanyl, atropine, or ephedrine); (4) extubating time (time from sugammadex administration till extubation); (5) postoperative pain score VAS score was assessed 2 h after extubation then every 6 h for 48 h; (6) ICU and hospital length of stay (max 28 days) defined as the number of days after extubation before first hospital discharge [Time Frame: 28 Days]; (7) surgical time from skin incision to closure; (8) anaesthesia time from induction to extubation; (9) hemodynamic heart rate, blood pressure, cardiac output, systemic vascular resistance.

### Calculation of sample size

The sample size was calculated using G*Power 3 (Heinrich Heine University, Dusseldorf, Germany). Based on an internal pilot study of 24 patients divided into two equal groups. A total number of 92 cases divided into two equal groups was necessary to achieve the power of 90% assuming an alpha level of 0.05 (effect size d = 0.695) and independent samples t-test for inferential statistics. The sample size was increased to 100 cases to compensate for possible dropouts.

### Statistical analysis

Statistical analysis was performed using SPSS (Statistical Package for the Social Sciences) version 20, IBM Corp., U.S.A. The Kolmogorov-Smirnov test was used to examine data distribution. The Fischer exact or Chi-square test of independence was used to show relationships between binary variables. According to data distribution either independent samples student t-test or Mann-Whitney-U test were used to compare the two studied groups of quantitative normally distributed and skewed data, respectively. In all tests, results were considered statistically significant if the P- value was less than 0.05.

## Results

### Comparison of patient characteristics data

**Fig. 1:**
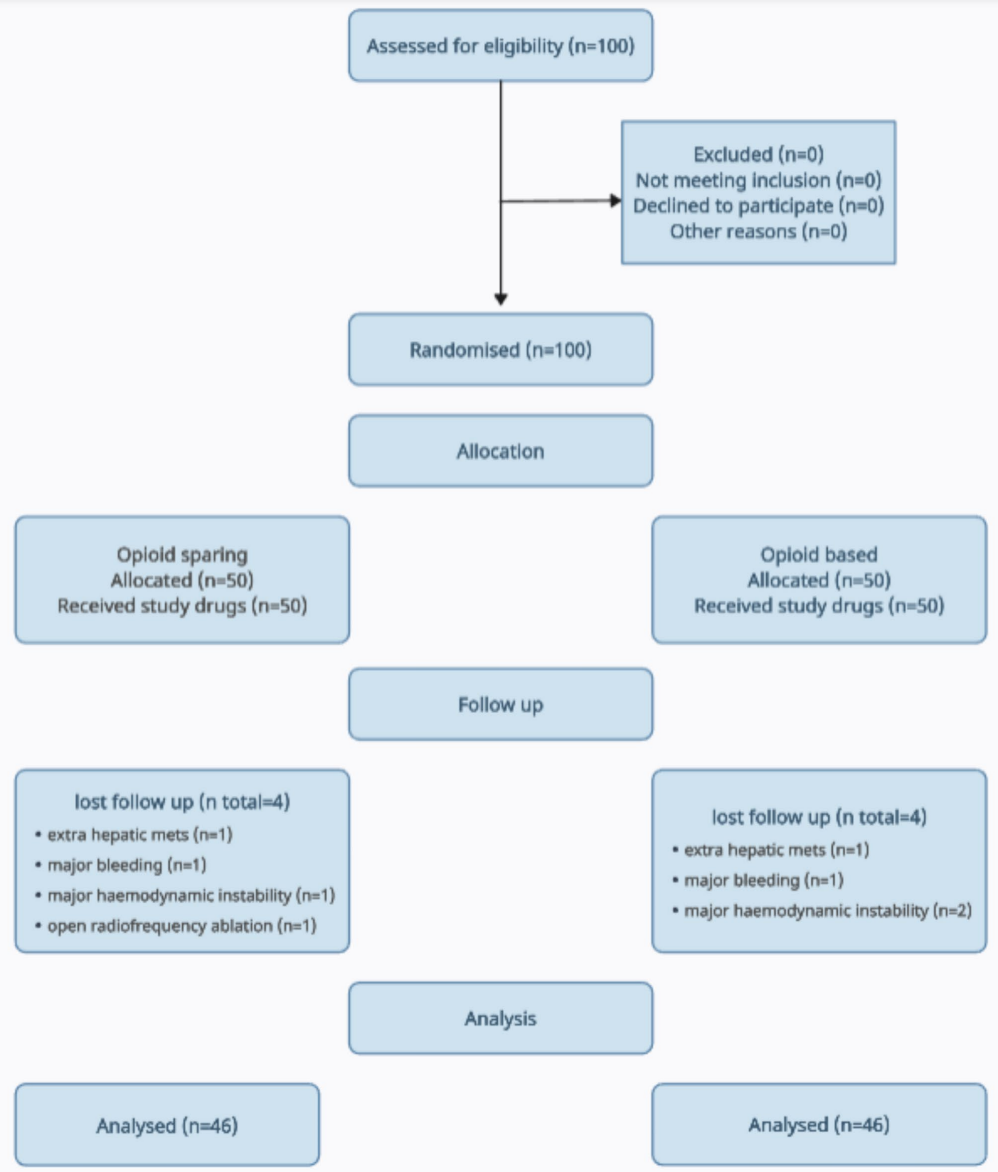
CONSORT flow diagram showing patients allocation at different stages of the study. Mets, metastasis

Ninety-two patients completed the study: 46 patients in the OS group and 46 patients in the OB group. Eight patients dropped out (Fig. 1). The average age, weight, and gender distribution were comparable in the two study groups (Table 1).

**Table 1:** Patient characteristics. Values are mean ± SD or number

Variable	Group 1 Opioid sparing (OS) (*n* = 46)	Group 2 Opioid based (OB) (*n* = 46)	*P* value
Age (years)^1^	55.1 ± 8.22	58 ± 5.86	0.06
Weight (kg)^1^	78.8 ± 9.17	79.2 ± 8.3	0.81
Gender (M/F)	33/13	34/12	
Liver resection			
Formal right lobe resection	12	12	
Left lateral resection	10	12	
Non-anatomical resection	23	18	
Formal left lobe resection	0	4	
Wedge resection	1	0	

1: Data were normally distributed ; tested by T-test

### Comparison of intra and post operative anesthetics between the two groups

The total intraoperative and 1st 48 h of postoperative fentanyl consumption was significantly lower in the opioid-sparing group. The average end-tidal desflurane was significantly lower in the opioid-sparing group.

### Comparison recovery, ICU and hospital stays among groups

Anaesthesia, surgical, and extubating times were significantly shorter in the opioid-sparing group. Although the ICU stay was significantly shorter in the opioid-sparing group, the hospital stay was comparable between the two studied groups (Table 2).

**Table 2:** Intra-operative and ICU fentanyl dose, operative times, ICU and hospital stay. Values are mean ± SD

Variable	Group 1 Opioid sparing (OS) (*n* = 46)	Group 2 Opioid based (OB) (*n* = 46)	*P* value
Total intra-operative fentanyl dose (µg)	183.2 ± 35.61	313.5 ± 75.06	˂ 0.001*
Total 1st 48 h ICU fentanyl dose (µg)	354.5 ± 112.62	779.1 ± 294.97	˂ 0.001*
Average end-tidal desflurane (%)	5.3 ± 0.85	8.3 ± 0.70	˂ 0.001*
Duration of anaesthesia (min)	213 ± 86.16	261.1 ± 81.46	0.007 *
Duration of surgery (min)	178.3 ± 81.57	220.5 ± 77.02	0.012 *
Extubation time (min)	10.2 ± 2.69	15.1 ± 3.02	˂ 0.001 *
ICU stay (day)	1.3 ± 0.60	2.1 ± 1.24	0.005 *
Hospital stay (day)	4.9 ± 1.91	6.0 ± 2.61	0.056

* = Significant difference between Opioid sparing (OS) and Opioid based (OB)

ICU = intensive care unit

1 =data were normally distributed tested by T-test

2 = data were not normally distributed tested by Mann-Whitney test.

### Hemodynamics in the studied groups

Episodes of bradycardia were comparable between the two studied groups. Intra-operative hypotension events were significantly more frequent in the opioid-sparing group, leading to significantly higher total rescue ephedrine dose and number of doses. Intra-operative hypertension events were significantly more frequent in the opioid-based group. The total rescue fentanyl dose and number of doses were significantly higher in the opioid-based group (Table 3).

**Table 3:** Intraoperative hemodynamic events and rescue drugs. Values are mean ± SD

Variable	Group 1 Opioid sparing (OS) (*n* = 46)	Group 2 Opioid based (OB) (*n* = 46)		*P* value	
Bradycardia incidence intraoperative	6.5%		17.4%	0.11	
Hypotension incidence intraoperative	100%		78.3%	˂ 0.001*	
Hypertension incidence intraoperative	26.1%		100%	˂ 0.001*	
Number of rescue fentanyl doses	0.35 ± 0.48		1.9 ± 0.84	˂ 0.001*	
Total rescue fentanyl dose (µg)	25.5 ± 35.98		155.0 ± 69.17	˂ 0.001 *	
Number of rescue ephedrine doses	2.4 ± 1.02		1.7 ± 1.24	0.006 *	
Total rescue ephedrine dose (mg)	12.1 ± 5.01		8.6 ± 6.18	0.006 *	

* = Significant difference between Opioid sparing (OS) and Opioid based (OB)

All variables data were not normally distributed tested by Mann-Whitney test

### The studied postoperative adverse events

postoperative desaturation, nausea, and vomiting were significantly more frequent in the opioid-based group. No patients developed ileus in the two groups. The postoperative visual analogue score was significantly lower in the opioid-sparing group in the early postoperative period at the second-hour assessment point. Then, pain scores were comparable at the subsequent assessment points (Table 4).

**Table 4:** Postoperative 1st 24 h VAS. Values are mean ± SD

Variable	Group 1 Opioid sparing (OS) (*n* = 46)	Group 2 Opioid based (OB) (*n* = 46)	*P* value
VAS 1 (2 h)	1.9 ± 0.77	2.9 ± 0.49	˂ 0.001*
VAS 2 (8 h)	2.4 ± 0.65	2.7 ± 0.58	0.08
VAS 3 (14 h)	2.7 ± 0.75	2.7 ± 0.68	0.73
VAS 4 (20 h)	2.3 ± 0.64	2.1 ± 0.94	0.26

* = Significant difference between Opioid sparing (OS) and Opioid based (OB)

VAS = Visual Analogue Scale hr. = hours post extubation

All variables data were not normally distributed tested by Mann-Whitney test

## Discussion

In our study, we observed significant reductions in total fentanyl consumption during the intra-operative and postoperative periods in the opioid-sparing group. Furthermore, the opioid-sparing group exhibited significantly lower postoperative pain scores, in the early postoperative period as assessed by the visual analogue scale at the 2nd hour following the extubation.

In a study conducted by Naik and colleagues [16], the opioid-sparing potentials of dexmedetomidine were examined in patients undergoing major spine surgery. Although the use of dexmedetomidine during the intra-operative period resulted in a reduction in opioid administration, the study was terminated early after an interim analysis and failed to demonstrate that intraoperative dexmedetomidine could effectively reduce postoperative opioid consumption or improve pain scores in patients undergoing multilevel deformity correction spine surgery.

While our study showed an intra-operative opioid-sparing effect consistent with the findings of the Naik et al. study, the results differed regarding postoperative opioid-sparing effects. This discrepancy could potentially be attributed to the use of methadone as the intra-operative opioid in the Naik et al. study [16]. Methadone is known for its long half-life and NMDA receptor antagonist properties [17]. This may have contributed to masking the postoperative opioid-sparing effects of dexmedetomidine. In our study, fentanyl was administered as the intra-operative analgesic, and we also utilized intra-operative ketamine infusion. It is worth noting that ketamine has demonstrated preventive analgesic effects extending beyond its clinical duration of action [18]. 

A systematic review conducted by Lundorf and coworkers [19] on the peri-operative effects of dexmedetomidine on acute pain after abdominal surgery, concluded that dexmedetomidine administered during the peri-operative period has some opioid-sparing effect. Additionally, the review found that there were generally no significant differences in postoperative pain scores between the dexmedetomidine group and other comparator groups. In contrast to this finding, our study revealed significantly lower postoperative pain scores in the early postoperative period, as measured by the visual analogue scale, at the 2nd hour following the extubation.

In a systematic review and meta-analysis of randomized controlled trials conducted by Blaudszun and colleagues [20], the effect of peri-operative systemic alpha-2 agonists on postoperative morphine consumption and pain intensity was investigated. The study findings indicated that dexmedetomidine exhibited a morphine-sparing effect at 2 h, 12 h, and 24 h postoperatively. Furthermore, the use of dexmedetomidine was associated with a significant reduction in postoperative pain intensity, as evidenced by reductions in visual analogue scale (VAS) pain scores at 2 h and 24 h.

Although the previous systematic reviews focused exclusively on peri-operative dexmedetomidine without incorporating ketamine, their findings are generally in line with our study findings in terms of postoperative opioid-sparing effects and improved pain scores. These reviews consistently demonstrated that dexmedetomidine administration during the peri-operative period led to reduced opioid consumption and better pain control. Despite the absence of ketamine in their analyses, their results support the notion that dexmedetomidine can effectively contribute to opioid-sparing and improved postoperative pain management.

In a retrospective study conducted by Halaszynski and colleagues [21], the potential of peri-operative ketamine to enhance analgesia in donor hepatectomy surgery was investigated. The study reported that the administration of ketamine during the peri-operative period resulted in improved analgesia and reduced opioid requirements compared to the use of patient-controlled analgesia (PCA) opioids alone.

In a review and meta-analysis conducted by Wang and coworkers [22], they found moderate to low evidence supporting the postoperative opioid-sparing effects of peri-operative ketamine for up to 12 h. Additionally, ketamine administration led to significant improvements in pain scores for up to 24 h after surgery. Similarly, a systematic review by Subramaniam and colleagues [23], highlighted that a low dose of ketamine as an adjuvant analgesic to opioids resulted in significant opioid-sparing effects, especially in procedures with high opioid requirements. However, while ketamine showed promise in reducing opioid consumption, there was insufficient evidence to support a reduction in opioid-related adverse events through its administration. Overall, these findings suggest that ketamine has the potential to enhance postoperative pain management and reduce opioid usage within specific time frames.

Previous studies have demonstrated that peri-operative administration of ketamine alone can effectively provide opioid-sparing effects [24]. However, when ketamine is combined with dexmedetomidine, a synergistic analgesic effect can be achieved as they act on different sites of action [25]. The combination of ketamine and dexmedetomidine could be associated with several advantages, including hemodynamic stability, absence of respiratory depression, improved postoperative analgesia, and enhanced recovery [26]. This combination has been commonly used for procedural sedation in both adult and pediatric patients, sedo-analgesia during burn dressing procedures, and as an adjunct to minor procedures in various clinical settings [27–31]. 

In their study, Priya Thappa and co-authors [32] explored the impact of combining intra-operative ketamine and dexmedetomidine on postoperative analgesic requirements in spine surgeries. Their findings indicated that the intra-operative infusion of ketamine and dexmedetomidine could completely replace intra-operative fentanyl in spine surgeries. In our study focusing on major abdominal surgeries like liver resection, we observed a reduction of approximately 40% in intra-operative fentanyl requirements.

Concerning postoperative fentanyl usage, the infusion of ketamine and dexmedetomidine demonstrated a substantial opioid-sparing effect amounting to approximately 38% in spine surgeries and approximately 55% in our investigation. Additionally, analysis of pain scores in both studies revealed a noteworthy reduction in pain scores during the early postoperative period.

Baseline hemodynamic were similar across our study groups. Episodes of bradycardia were statistically similar between the two groups. Interestingly, the incidence of bradycardia was paradoxically 2.5 times more frequent in the opioid-based group. This might be attributed to the effects of increased intra-operative fentanyl consumption [33]. On the other hand, it is noteworthy that ketamine mitigates the bradycardic effects of dexmedetomidine [26]. Intra-operative hypotension events occurred more frequently in the opioid-sparing group, resulting in increased ephedrine consumption. Every patient in the opioid-sparing group experienced at least one hypotensive episode requiring a rescue dose of ephedrine. In their investigation aiming to discern the causes and patterns of hemodynamic shifts during hepatic resection surgery, Gelmanas and colleagues [34] observed hypotensive episodes. Among the 55 patients studied, hypotension occurred in 53 patients, totaling 186 episodes, with an average of 3.4 episodes per patient (standard deviation, 2.0). Hypotension emerged as a predominant hemodynamic alteration during hepatic surgery, highlighting its significance in this clinical setting.

The lower intraoperative and postoperative fentanyl consumption in the opioid sparing group suggests effective reduction of opioid use while maintaining pain control, crucial amidst the opioid crisis. Shorter anesthesia, surgical, and extubation times indicate faster recovery, potentially reducing healthcare costs and enhancing operational efficiency. Lower rates of postoperative respiratory complications, nausea and vomiting in the opioid sparing group could improve patient comfort and satisfaction. Early postoperative pain management was more effective in the opioid sparing group, contributing to enhanced patient comfort. The shorter ICU stays with opioid sparing anesthesia suggests quicker recovery and potentially lower healthcare costs. These findings collectively highlight the benefits of opioid sparing anesthesia in reducing opioid consumption, improving recovery outcomes, enhancing pain management, and minimizing postoperative complications, guiding clinicians towards optimized patient care strategies.

The observed effects of opioid sparing anesthesia compared to opioid-based anesthesia in the study may be attributed to various potential mechanisms. Utilizing a multimodal analgesic approach in opioid sparing strategies targets multiple pain pathways, enhancing pain relief while minimizing opioid requirements. By preserving the endogenous opioid system function and avoiding excessive exogenous opioid exposure, opioid sparing techniques may promote the release of endogenous opioids, improving pain modulation. Mitigating neuroplastic changes in the central nervous system through reduced opioid exposure could prevent central sensitization and improve long-term pain outcomes. Reduced suppression of the immune system and inflammatory response with opioid sparing anesthesia could lead to faster recovery and decreased postoperative complications.

This study has some limitations: reliance on subjective measurements of hemodynamic for evaluating intra-operative pain and determining fentanyl administration, suggesting a need for objective pain assessment through methods like the Analgesia Nociception Index (ANI), absence of long-term follow-up of study groups to evaluate the incidence of chronic pain and long-term complications associated with opioids, such as tumor recurrence, the Minimum Alveolar Concentration (MAC) of desflurane varied between the two study groups. Desflurane’s analgesic properties could have potentially influenced opioid requirements, introducing a confounding factor, and the incidence of postoperative delirium not assessed in our patients.

## Conclusion

Administering dexmedetomidine and ketamine infusions intra-operatively to patients with Child A liver cirrhosis undergoing liver resection resulted in notable opioid-sparing effects, with reductions of approximately 40% intra-operatively and 55% postoperatively. The opioid-sparing group exhibited improved postoperative outcomes, including reduced pain in the early postoperative period, decreased incidence of postoperative nausea and vomiting and shorter ICU stays.

## Electronic supplementary material

Below is the link to the electronic supplementary material.

Supplementary Material 1

## Data Availability

The datasets used and/or analyzed during the current study available from the corresponding author on reasonable request.the data present in excel sheet .

## Declarations

### Clinical registration

The study was registered in the ClinicalTrials.gov identifier number: NCT05674877.

## Abbreviations

OS: Opioid-sparing group
OB: Opioid-based (OB) group
ICU: Intensive care unit
BIS: Bispectral index
EC: electrical cardiometry
CVP: Central Venous Pressure
PCA: Patient control analgesia
TAP: Transversus abdominis plane
VAS: Visual analogue scale
ASA: American Society of Anesthesiologists
ANI: Analgesia Nociception Index
