# Supplementary material for: Opioid sparing anesthesia in patients with liver cirrhosis undergoing liver resection: a controlled randomized double-blind study
Source: BMC Anesthesiol. 2025 Feb 1;25:53. doi: 10.1186/s12871-025-02915-4 (PMC11786452; doi:10.1186/s12871-025-02915-4)
Supplement: Supplementary file 1 — Supplementary Material 1 [file 12871_2025_2915_MOESM1_ESM.docx]

**Trial Protocol**

**OPIOID SPARING ANESTHESIA in PATIENTS with LIVER CIRRHOSIS UNDERGOING LIVER RESECTION: A CONTROLLED RANDOMIZED DOUBLE-BLIND STUDY.**

**Inclusion and exclusion criteria**

Ethical approval for this study (Ethical Committee N° 00309/2022) was provided by the Institutional Review Board of the National Liver Institute, Menoufia University (Chairperson Prof Azza. Abd Elaziz) on 31 July 2022. The study was registered in the ClinicalTrials.gov identifier number: NCT05674877. The study was conducted in the Anesthesiology Department, National Liver Institute between August 2022 and July 2023. Written informed consent was taken from each patient. The study included adult patients with Child class (A) liver cirrhosis aged 18 to 65 years undergoing liver resection.

**Randomization and blind**

Patients were randomly allocated into one of the two study groups, the opioid-sparing group (OS) and the opioid-based group (OB) using an online randomization program (<http://www.randomizer.org>). Random allocation numbers were concealed in opaque closed envelopes.

**Anesthesia technique**

Patients fulfilling the inclusion criteria underwent clinical evaluation including preoperative laboratory assessment of liver and renal functions the day before surgery. Other diagnostic or laboratory workup was requested by the attending anesthesiologist and the surgeon according to the patient clinical condition. We excluded patients with renal or cardiac dysfunction, a history of chronic pain, alcohol or drug abuse, analgesic use in the last 24 hours before surgery, major intraoperative hemodynamic instability, the need for postoperative ventilation, inability to comprehend pain assessment and allergy or contraindication to any of the study medications.

Basic intraoperative monitoring included: electrocardiography, pulse oximetry, end-tidal CO_2_, invasive arterial blood pressure, central venous pressure, electrical cardiometry (EC) (ICON monitor; Cardiotronics Inc., La Jolla, CA, USA), esophageal temperature, fraction inspired oxygen, expired end-tidal desflurane concentration, and urine output. Depth of anaesthesia was monitored using Bispectral index, and neuromuscular function with TOF-Watch SX (Schering-Plough, Swords, Co. Dublin, Ireland).

The success and spread of the ultrasound-guided bilateral transversus abdominis plane (TAP) blocks were assessed in all patients by the pinprick test after administering 20 ml of 0.25% levobupivacaine on each side before induction of anaesthesia. Anaesthesia was induced in all patients using fentanyl 2µg kg^-1^, propofol 2 mg kg^-1^, and rocuronium 0.6 mg kg^-1^ to facilitate endotracheal intubation.

**Interventions**

After induction of anaesthesia, patients were randomly allocated in two groups opioid sparing group (OS) and opioid-based group (OB). Patients in the opioid-sparing group received a loading dose of dexmedetomidine (1 µg kg^-1^ over 10 minutes). This was followed by a fixed continuous maintenance infusion of 0.5 µg kg^-1^h^-1^. Furthermore, a single induction analgesic dose of 0.5 mg kg^-1^ ketamine was given to all patients in the OS group. This was followed by 0.25 mg kg^-1^ h^-1^ continuous maintenance infusion. Dexmedetomidine and ketamine infusions were stopped 30 minutes before the conclusion of surgery. Patients in the opioid-based group received placebo-equivalent boluses and infusions of 0.9 % saline. The attending anesthesiologist was blinded to the patient group assignment.

Anaesthesia was maintained with air, oxygen, and desflurane to keep a BIS value between 40 and 60. Muscle relaxation was maintained by additional top-up doses of rocuronium

0.15mg kg^-1^ and was guided by the response to ulnar nerve stimulation. Ventilation parameters were adjusted to maintain normocapnia. Intraoperative normothermia was maintained using a forced air warm blanket (Model 750-Bair Hugger Temperature Management Unit, SMA MISR, Arizant Healthcare Inc, USA), a humidifier, and warm intravenous fluids. Deep venous thrombosis (DVT) prophylaxis included elastic stockings and, sequential compression device (SCD) (Kendall Company, Tyco, USA) on the lower limb until early ambulation. Intraoperative fluid, fresh frozen plasma, and blood replacement therapy were guided by the continuous monitoring of the central venous pressure and EC Cardiometry and were titrated to maintain hemodynamic stability and a hemoglobin level of 10 g dL^-1^.

The intra-operative hemodynamic target was the mean arterial blood pressure and heart rate within 20% of the baseline value. Significant hemodynamic alterations were managed as follows: bradycardia (heart rate <50 beats min^-1^) was managed by incremental 0.5 mg doses of atropine, hypertension and or tachycardia defined as more than 20% increase of the baseline readings was managed by top-up doses of fentanyl 1µg kg^-1^ in the two study groups, hypotension defined as more than 20% reduction in the baseline mean arterial blood pressure was managed by incremental doses of ephedrine 5mg in the two study groups.

At the end of surgery and when two responses to train-of-four ulnar nerve stimulation were detected (T2), residual rocuronium-induced neuromuscular block was antagonized by sugammadex 2 mg kg^-1^. Patients were extubated and discharged to the surgical intensive care unit after achieving a train-of-four ratio of 0.9. Postoperative analgesia was achieved using patient-controlled fentanyl infusion (PCA fentanyl).

**Study outcomes**

The Primary outcome measure was the Intra-operative fentanyl requirements. The Secondary outcome measures included: 1) postoperative PCA fentanyl requirements over the first 48 hours postoperatively; 2) incidence of severe postoperative opioid-related adverse events as desaturation episodes (on room air), postoperative nausea and vomiting and postoperative ileus [time Frame: 48 hours after extubation. Desaturation is a decrease of oxygen saturation equal to or exceeding 4% of the baseline value. Postoperative ileus is defined as the absence of flatus or stools within the first 48 hours after extubation; 3) incidence of bradycardia, hypotension, and hypertension events during surgery and the number and doses of rescue medications during surgery (fentanyl, atropine, or ephedrine); 4) extubating time (time from sugammadex administration till extubation); 5) postoperative pain score VAS score was assessed 2 hours after extubation then every 6 hours for 48 hours; 6) ICU and hospital length of stay (max 28 days) defined as the number of days after extubation before first hospital discharge [Time Frame: 28 Days]; 7) average required end-tidal desflurane concentration; 8) surgical time from skin incision to closure; 9) anaesthesia time from induction to extubation; 10) hemodynamic heart rate, blood pressure, cardiac output, systemic vascular resistance.

**Calculation of sample size**

The sample size was calculated using G*Power 3 (Heinrich Heine University, Dusseldorf, Germany). Based on an internal pilot study of 24 patients divided into two equal groups. A total number of 92 cases divided into two equal groups was necessary to achieve the power of 90% assuming an alpha level of 0.05 (effect size d = 0.695) and independent samples t-test for inferential statistics. The sample size was increased to 100 cases to compensate for possible

dropouts.
